# The Association of *CYP19A1* Variation with Circulating Estradiol and Aromatase Inhibitor Outcome: Can *CYP19A1* Variants Be Used to Predict Treatment Efficacy?

**DOI:** 10.3389/fphar.2017.00218

**Published:** 2017-04-25

**Authors:** Dylan M. Glubb, Tracy A. O'Mara, Jannah Shamsani, Amanda B. Spurdle

**Affiliations:** Molecular Cancer Epidemiology, Department of Genetics and Computational Biology, QIMR Berghofer Medical Research InstituteBrisbane, QLD, Australia

**Keywords:** *CYP19A1*, estradiol, genetic variation, aromatase inhibitors, patient outcome, breast cancer, endometrial cancer, pleiotropy

## Abstract

After menopause, estradiol is primarily synthesized in peripheral tissues by the enzyme aromatase, encoded by *CYP19A1*. *CYP19A1* variation associates with circulating estradiol in postmenopausal women and this variation is best represented by the intronic variant rs727479. This variation appears to have pleiotropic effects as it also associates with endometrial cancer risk. Indeed, estradiol plays an important role in the development of breast and endometrial cancer. Aromatase inhibitor (AI) drugs are used in the treatment of both diseases, however, response rates for AIs are low and there is currently no way to identify breast or endometrial cancer patients who are more likely to receive a clinical benefit. Multiple studies have proposed that genetic variation in *CYP19A1* will have effects on AI efficacy: eight candidate variant studies of sample size greater than 50 describe associations between *CYP19A1* variation and the outcome of patients treated with AIs. Nominally significant associations with patient outcome were reported for several variants, including rs727479. However, only an association between rs4646 and time to progression was replicated in an independent study. Moreover, rs4646 is also the only variant that has an association with patient outcome that passes a multiple testing threshold and this variant is in linkage disequilibrium with rs727479, supporting the hypothesis that associations with patient outcome may be driven through the effects on circulating estradiol. Despite this preliminary evidence, well phenotyped and comprehensively genotyped patient sets need to be studied before conclusions can be drawn about the effects of *CYP19A1* variation on AI efficacy.

## Introduction

Estrogen exposure is strongly associated with breast and endometrial cancer carcinogenesis (Setiawan et al., 2013; Anderson et al., 2014). In the biosynthesis of estrogens, aromatase is the final and rate-limiting enzyme in humans (reviewed in Bulun et al., 2005; Zhao et al., 2016). In premenopausal women ovaries have the highest levels of aromatase, while in postmenopausal women aromatase is found at greatest concentrations in peripheral tissues such as adipose (Bulun et al., 2005). Increased aromatase activity may be responsible for the higher circulating estrogen levels that are observed in postmenopausal breast and endometrial cancer (Austin et al., 1991; Nyholm et al., 1993; Zhang et al., 2013). Indeed, we and other groups have identified associations between estradiol levels and *CYP19A1* variation (Haiman et al., 2007; Beckmann et al., 2011; Prescott et al., 2012; Thompson et al., 2016). We have also observed that circulating estradiol and genetic variation in *CYP19A1*, the gene encoding aromatase, have similar effects on endometrial cancer risk (Thompson et al., 2016), suggesting that this variation mediates its effects by up-regulating *CYP19A1* expression. Moreover, *CYP19A1* variation may have clinical implications for anti-estrogen therapies used in the treatment of breast and endometrial cancer.

Therapies targeting the estrogen pathway in breast and endometrial cancer include tamoxifen, whose metabolites inhibit estrogen receptor activity, and aromatase inhibitors (AIs) such as anastrozole, letrozole, and exemestane which ultimately act by reducing circulating estrogen. Although tamoxifen acts as an antagonist of the estrogen receptor in breast cancer, in the endometrium it has an estrogenic effect and stimulates signaling through the estrogen receptor (reviewed in Hu et al., 2015). Consequently, tamoxifen is not used to treat primary endometrial cancer and its usage in the treatment of early stage breast cancer is associated with increased endometrial cancer risk (Howell et al., 2005; Cuzick et al., 2010). In contrast, there are promising results for the use of AIs in early endometrial cancer (Berstein et al., 2002, 2005; Barker et al., 2009) and their efficacy in early breast cancer is well established (Ellis et al., 2001; Ellis and Ma, 2007; Cuzick et al., 2010; Regan et al., 2011). Nevertheless, ~45% of early-stage breast cancer patients with hormone-receptor positive (ER+ and progesterone receptor+) disease do not respond to AIs (Ellis et al., 2001; Ellis and Ma, 2007) and up to 30% of patients will experience disease recurrence (Cuzick et al., 2010). AIs are also used to treat advanced breast cancer but, again, around 50% of patients do not respond (Gershanovich et al., 1998; Mouridsen et al., 2001; Paridaens et al., 2008). Response rates are much worse for advanced endometrial cancer, with only ~9% of patients responding to AI therapy (Rose et al., 2000; Ma et al., 2004; Lindemann et al., 2014). Markers that predict AI efficacy are thus sorely needed and could improve patient outcomes by identifying the patients who are more likely to benefit from this therapy.

This review assesses *CYP19A1* variation associated with estrogen levels to determine if correlated variation is also associated with patient outcomes after AI therapy. To this end, we firstly summarize results from studies in postmenopausal women that have investigated the association of *CYP19A1* variation with circulating estradiol, the most biologically active estrogen (Files et al., 2011). Secondly, we critically review findings from clinical trials that have tested the association of *CYP19A1* variation with patient outcomes. Lastly, we assess variation associated with patient outcomes for correlation with variation associated with circulating estradiol levels.

## *CYP19A1* variation and association with circulating estrogen levels in postmenopausal women

Due to the role of aromatase in estrogen biosynthesis, many studies have tested associations between *CYP19A1* genetic variation and circulating estradiol levels in postmenopausal women. However, to gain a clearer picture of the field, we have focused on the largest studies and included for review six studies that have analyzed more than 800 women. All of these studies predominantly include women of European ancestry and the main findings identify significant associations with eight *CYP19A1* variants (Table 1). In the four studies in which either genome-wide, imputation or tagging SNP analyses were used (as opposed to a candidate variant approach), rs727479, an intronic *CYP19A1* variant, had the strongest associations with circulating estradiol (Table 1). In all the reviewed studies, the effect of rs727479 was consistent i.e., the minor (C) allele of rs727479 was associated with lower estradiol levels (Table 1) with a decrease in estradiol of between 5 and 10.7% when comparing heterozygotes to major allele homozygotes (Haiman et al., 2007; Beckmann et al., 2011; Prescott et al., 2012; Thompson et al., 2016). Importantly, Thompson et al., used conditional analysis to demonstrate that there is a single genetic signal, represented by rs727479, that is associated with estradiol levels (Thompson et al., 2016). Indeed, all single nucleotide polymorphisms associated with estradiol levels share some degree of linkage disequilibrium with rs727479 in the 1,000 Genomes Project European populations (*r*^2^ = 0.10–0.51). Furthermore, rs727479 alters the binding motifs of four transcription factors and is located in a putative regulatory element within *CYP19A1* (Thompson et al., 2016). These findings suggest that rs727479 and/or genetic variation in strong linkage disequilibrium with this variant, has a causal effect on estradiol levels through regulation of *CYP19A1* expression.

**Table 1:** **Associations of ***CYP19A1*** variants and circulating estradiol in postmenopausal women**.

**Variant**	***CYP19A1* location (Chr15 position)**	**LD with rs727479**	**Study**	***N***	**Association with circulating estradiol**
rs60271534 (7, 8, 11, and 12 repeats)	Intronic (51502844)	N/A^*^	Haiman et al., 2000	1,236	No significant associations
rs730154 (A/G)	Intronic (51299007)	0.05 (EUR)	−	−	Not tested
rs936308 (C/G)	Intronic (51288877)	0.05 (EUR)	−	−	Not tested
rs749292 (G/A)	Intronic (51266534)	0.36 (EUR)	Haiman et al., 2007	3,391	Higher estradiol (*p* = 1.3 × 10^−10^) in A allele carriers
			Beckmann et al., 2011	2,721	Higher estradiol (*p* = 0.014) in A allele carriers
rs6493494 (G/A)	Intronic (51257638)	0.34 (EUR)	Haiman et al., 2007	3,431	Higher estradiol (*p* = 3.0 × 10^−8^) in A allele carriers
			Beckmann et al., 2011	2,721	Higher estradiol (*p* = 0.02) in A allele carriers
rs1008805 (A/G)	Intronic (51257402)	0.17 (EUR)	Haiman et al., 2007	3,385	Lower estradiol (*p* = 4.8 × 10^−6^) in G allele carriers
rs10459592 (G/T)	Intronic (51243944)	0.68 (EUR)	−	−	Not tested
rs4775936 (C/T)	Intronic (51243825)	0.44 (EUR)	−	−	Not tested
rs727479 (A/C)	Intronic (51242350)	1	Haiman et al., 2007	3,354	Lower estradiol (*p* = 6.4 × 10^−11^) in C allele carriers
			Beckmann et al., 2011	2,721	Lower estradiol (*p* = 0.001) in C allele carriers
			Prescott et al., 2012	1,583	Lower estradiol (*p* = 5.11 × 10^−7^) in C allele carriers
			Thompson et al., 2016	2,767	Lower estradiol (*p* = 7.4 × 10^−8^) in C allele carriers
rs2414096 (G/A)	Intronic (51237582)	0.51 (EUR)	Haiman et al., 2007	3,386	Higher estradiol (*p* = 1.0 × 10^−9^) in A allele carriers
rs700518 (A/G)	Exonic: synonymous (51236915)	0.51 (EUR)	−	−	Not tested
rs11575899 (TCT/−)	Intronic (51227749)	N/A^*^	Dunning et al., 2004	871	Lower estradiol (*p* = 0.0003) in deletion allele carriers
rs28757184 (C/T; Thr/Met)	Exonic: missense (51222375)	0.02 (EUR)	−	−	Not tested
rs700519 (C/T; Arg/Cys)	Exonic: missense (51215771)	0.02 (EUR)	−	−	Not tested
rs10046 (C/T)	3′UTR (51210789)	0.51 (EUR)	Dunning et al., 2004	1,747	Higher estradiol (*p* = 0.0006) in T allele carriers
			Haiman et al., 2007	3,325	Higher estradiol (*p* = 2.9 × 10^−9^) in T allele carriers
			Beckmann et al., 2011	2,721	Higher estradiol (*p* = 0.001) in T allele carriers
rs4646 (C/A)	3′UTR (51210647)	0.10 (EUR)	Haiman et al., 2007	3,431	Lower estradiol (*p* = 1.3 × 10^−3^) in A allele carriers

*GRCh38*.

* *Linkage disequilibrium data from the 1,000 Genomes Project are not available for these variants*.

## Effect of *CYP19A1* variation on patient outcome after AI therapy

We identified 10 association studies between *CYP19A1* variants and breast cancer patient outcomes after AI therapy, but we could find no such pharmacogenetic reports in endometrial cancer. After excluding small studies (<50 patients) with particularly low statistical power (and potentially poor quality), eight relevant studies were identified (Table 2); all but one of these studies (Park et al., 2011) was limited to postmenopausal women; and five of the eight studies analyzed data from patients with metastatic breast cancer, while the remaining three studies assessed patients with earlier stage disease.

**Table 2:** **Associations of ***CYP19A1*** variants and patient outcomes after AI therapy (***n*** > 50)**.

**Study**	**N (ethnicity)**	**Aromatase inhibitors (Dose)**	**Treatment setting**	**SNP**	**LD with rs727479 (r^2^)^§^**	**Primary endpoint**	**Outcome**		**HR/OR 95% CI)**	***P*-value**
							**Major allele**	**Minor allele**		
Gervasini et al., 2016	110 (Spanish)	Anastrozole	Postmenopausal stage I–III HR+	rs727479	1	Recurrence	AA: 0% of relapsed patients 38% of non-relapsed patients	CA/CC: 100% of relapsed patients 62% of non-relapsed patients	**N/A**	0.03^*^
				rs1008805	0.17 (EUR)		TT: 14% of relapsed patients 25% of non-relapsed patients	CT/CC: 86% of relapsed patients 75% of non-relapsed patients	HR (CT/CC): 1.67 (0.18–15.01)	0.54
				rs749292	0.36 (EUR)		TT: 0% of relapsed patients 16% of non-relapsed patients	CT/CC: 100% of relapsed patient 85% of non-relapsed patients	N/A	0.39
				rs730154	0.05 (EUR)		GG: 57% of relapsed patients 28% of non-relapsed patients	AG/AA: 43% of relapsed patients 72% of non-relapsed patients	HR (AG/AA): 0.73 (0.07–1.97)	0.22
Colomer et al., 2008	65 (European)	Adjuvant letrozole (2.5 mg/day)	Postmenopausal metastatic HR+	rs4646	0.10 (EUR)	TTP	CC: 196 days	AC/AA: 525 days	HR (AC/AA): 0.52 (0.28–0.94)	0.02
				rs10046	0.51 (EUR)		CC: 288 days	TC/TT: 500 days	–	0.3
				rs727479	1		TT: 370 days	GT/GG: 294 days	–	0.9
Garcia-Casado et al., 2010	95 (Spanish)	Neoadjuvant letrozole (2.5 mg/day)	Postmenopausal stage II–III ER/PR+	rs4646	0.10 (EUR)	PFS	CC: 86% with no progression	AC/AA: 51% with no progression	–	0.07
						RR4M	CC: 74% of responders 52% of non-responders	AC/AA: 26% of responders 48% of non-responders	–	0.03
				rs10046	0.51 (EUR)	PFS	CC: 80% with no progression	TC/TT: 81% with no progression	–	0.97
						RR4M	CC: 17% of responders 15% of non-responders	TC/TT: 83% of responders 85% of non-responders	–	0.78
Liu et al., 2013	272 (Chinese)	Adjuvant anastrozole: 1 mg/day	Postmenopausal Metastatic HR+	rs4646	0.75 (EAS)	TTP	CC: 13.5 months	AC/AA: 16.4 months	HR (AC/AA): 0.50 (0.27–0.93)	0.049
						OS	CC: 31.6 months	AC/AA: 37.3 months	**HR (CC): 2.37 (1.20–4.65)**	**0.001^#^**
				rs10046	0.36 (EAS)	TTP	CC: 16.9 months	TC/TT: 14.9 months	–	0.94
						OS	–	–	–	**NS**
Artigalás et al., 2015	337 (Chinese and European)	Adjuvant letrozole	–	rs4646	0.75 (EAS) 0.10 (EUR)	TTP	–	–	HR (AC/AA): 0.51 (0.33–0.78)	0.002
Park et al., 2011	109 (Korean)	Postmenopausal women: adjuvant letrozole: (2.5 mg/day)	Post and premenopausal metastatic ER/PR +	rs700518	0.30 (EAS)	CBR	TC: 34% of patients with CB 58% of patients with no CB	TT/CC: 66% of patients with CB 43% of patients with no CB	**OR (TT/CC): 2.52 (1.02–6.20)**	**0.044**
		Premenopausal women: letrozole combined with goserelin: 3.6 mg/month		rs10459592	0.30 (EAS)		GT: 33% of patients with CB 55% of patients with no CB	GG/TT: 67% of patients with CB 45% of patients with no CB	**OR (GG/TT): 2.61 (1.06–6.46)**	**0.038**
				rs4775936	0.29 (EAS)		CT: 32 of % patients with CB 55% of patients with no CB	CC/TT: 68% of patients with CB 45% of patients with no CB	**OR (CC/TT): 2.89 (1.16–7.22)**	**0.023**
Ferraldeschi et al., 2012	308 (British; 90% Caucasian)	Adjuvant anastrozole, letrozole or exemestane	Postmenopausal metastatic HR+	rs4775936	0.44 (EUR)	TTF	–	−	HR (T allele): 0.79 (0.66−0.95)	0.01
				rs60271534	N/A		−	−	HR (>7 repeats): 0.84 (0.70−0.99)	0.04
				rs10459592	0.68 (EUR)		−	−	HR (T allele):1.19 (1−1.43)	0.05
				rs4646	0.10 (EUR)		−	−	HR (A allele):1.08 (0.85−1.38)	0.52
	166	Adjuvant letrozole		rs4775936	0.44 (EUR)		−	−	HR (T allele): 0.79 (0.66−0.95)	0.01
				rs10459592	0.68 (EUR)		−	−	HR (T allele): 1.21 (0.95−1.54)	0.18
				rs4646 (C/A)	0.10 (EUR)		−	−	HR (AA/CA): 1.01 (0.72−1.41)	0.98
Leyland-Jones et al., 2015	1236 (98% white in overall study)	5-year letrozole (2.5 mg/day)	Postmenopausal HR+ early breast cancer	rs4646 (C/A)	0.10 (EUR)	BCFI	−	−	**HR (A allele): 1.00 (0.81–1.23)**	**NS**
	1084			rs10046 (C/T)	0.51 (EUR)		−	−	**HR (C allele): 0.93 (0.76**−**1.13)**	**NS**
	618			rs700518 (T/C)	0.51 (EUR)		−	−	**HR (C allele): 1.14 (0.81**−**1.62)**	**NS**
	829			rs700519 (G/A)	0.02 (EUR)		−	−	**HR (A allele): 0.57 (0.27**−**1.23)**	**NS**
	720			rs936308 (C/G)	0.05 (EUR)		−	−	**HR (G allele): 0.60 (0.36**−**1.02)**	**NS**
	875			rs28757184 (G/A)	0.02 (EUR)				**HR (A allele): 1.58 (0.85**−**2.92)**	**NS**

*HR, hormone receptor; ER, estrogen receptor; PR, progesterone receptor; LD, linkage disequilibrium; RR4M radiological response at 4 months; DFS disease free survival, CBR clinical benefit rate; TTF, time to treatment failure; BCFI, breast cancer free interval*.

§ *Linkage disequilibrium data are from a corresponding 1,000 Genomes Project population (EUR, European; EAS, East Asian) but data are not available (N/A) for all variants*.

# *P-value passes Bonferroni threshold for multiple testing*.

* *Results from multivariate analyses are bolded*.

*Meta-analysis of Colomer et al. (2008) and Liu et al. (2013)*.

The SNP tagging the genetic variation with apparent causal effects on estradiol levels, rs727479, has been tested in only two of the reviewed studies, while an additional 13 *CYP19A1* variants, with varying degrees of linkage disequilibrium with rs727479 (*r*^2^ = 0.02–0.75 in European and East Asian 1,000 Genomes Project populations), have also been assessed (Table 2). Gervasini et al. tested rs727479 and three other *CYP19A1* variants for association in 110 Spanish stage I–III breast cancer patients of whom only seven patients had experienced recurrence (Gervasini et al., 2016). Of the four variants, only the C allele of rs727479 (correlated with lower circulating estradiol) was nominally associated with recurrence in both univariate analysis and multivariate analysis adjusted for patient and clinical characteristics. However, taking into account multiple testing, this association does not pass a stringent Bonferroni threshold for significance (*p* < 0.0125). Thus, it will be crucial to follow up these patients in the future to see if this association passes this threshold once more events have occurred. In another study, Colomer et al. assessed associations between rs727479 (and two other *CYP19A1* variants) and time to progression in 65 European metastatic breast cancer patients treated with adjuvant letrozole (Colomer et al., 2008). In univariate analyses, there was no significant association between time to progression and rs727479 or with correlated SNP rs10046 (*r*^2^ = 0.51). However, the A allele of rs4646, which has weak linkage disequilibrium with rs727479 (*r*^2^ = 0.10), was associated with longer TTP in univariate and multivariate analyses. Results from multivariate analyses for rs727479 and rs10046 were not reported.

The association between the A allele of rs4646 and longer time to progression (Colomer et al., 2008) reported by Colomer et al. was replicated by Liu et al. in univariate and multivariate analyses of 272 Chinese metastatic breast cancer patients treated with adjuvant letrozole (Liu et al., 2013). Notably, there was an association between the A allele of rs4646 and longer overall survival (OS) after multivariate analysis that passes a Bonferroni significance threshold for multiple testing (*p* < 0.025). In terms of molecular function, the effects of rs4646 have not been explored; however, as this variant is located in the 3′UTR of *CYP19A1*, it is possible that it may alter translation efficiency or mRNA stability. Alternatively, given in East Asians rs4646 is more strongly correlated with rs727479 (*r*^2^ = 0.75), the association with OS could be driven by rs727479 or other correlated variants.

A meta-analysis of the Colomer et al. (European) and Liu et al. (Chinese) studies revealed a significant overall association between rs4646 and TTP (*p* = 0.002) (Artigalás et al., 2015). However, no associations were observed between rs4646 and either time to treatment failure in a univariate analysis of 308 British metastatic breast cancer patients treated with adjuvant letrozole, anastrozole, or exemestane (Ferraldeschi et al., 2012), breast cancer free interval in a multivariate analysis of 1,236 predominantly white early stage breast cancer patients treated with letrozole (Leyland-Jones et al., 2015), or progression-free survival in 95 Spanish stage II–III breast cancer patients treated with neoadjuvant letrozole (Garcia-Casado et al., 2010). Further analysis of the Spanish stage II–III breast patients indicated that women carrying an A allele of rs4646 had worse tumor response after 4 months but associations were not adjusted for patient or clinical characteristics (Garcia-Casado et al., 2010). Moreover, this study uses response after 4 months rather than progression as an endpoint, making it difficult to interpret the inconsistency with the reports by Colomer et al. and Liu et al. which showed an association of better patient outcome with the A allele. Comparisons between these studies are further complicated by the use of different endpoints and treatment settings (i.e., neoadjuvant vs. adjuvant treatment; metastatic vs. earlier stage disease).

Three further *CYP19A1* variants have been reported to be associated with response to adjuvant letrozole. Park et al. (2011) found rs4775936, rs700518 and rs10459592 were nominally associated with clinical benefit rate after adjustment for patient and clinical characteristics in 109 post and premenopausal Korean breast cancer patients treated with letrozole. However, this study used an over-dominance model and compared associations between homozygotes (of either allele) and heterozygotes. Interpreting associations with the homozygote genotypes for these variants is difficult as the authors provided no rationale for using this genetic model. Two of these variants have also been tested in other studies with mixed results. rs700518 was analyzed in the Breast International Group 1–98 study, but was not associated with breast cancer-free interval in patients treated with letrozole (Leyland-Jones et al., 2015). Ferradelschi et al. identified an association between rs4775936 and time to treatment failure in British breast cancer patients treated with adjuvant AIs but this association was not significant after adjustment for patient and clinical characteristics (Ferraldeschi et al., 2012). Ferradleschi et al. also observed an association between rs60271534, an intronic short tandem repeat in *CYP19A1*, and time to treatment failure that lost significance after multivariate analysis.

## Discussion

rs727479 best represents the *CYP19A1* genetic variation that associates with circulating estradiol, but only two small studies of AIs have directly genotyped this variant: Gervasini et al. reported nominal association with recurrence (110 patients) (Gervasini et al., 2016), while Colomer et al. found no association with time to progression (65 patients) (Colomer et al., 2008). Comparisons between the studies are hampered by their heterogeneous nature. For example, patients of different disease stages (stage I–III vs. stage IV), have been treated with different AIs (anastrozole vs. letrozole), and different clinical endpoints (recurrence vs. TTP) measured. These features make it difficult to draw conclusions for associations of rs727479 with patient outcome.

Review of the literature reveals a lack of correction for multiple testing. Indeed, only one finding passed a Bonferroni threshold for significance: the association of rs4646 and overall survival in Chinese metastatic breast cancer patients treated with letrozole (Liu et al., 2013). Interestingly, rs4646 is the variant which is most strongly correlated with rs727479 (in the 1,000 Genomes Project East Asian populations). Moreover, the association of rs4646 with breast cancer patient outcome is the only finding that has been replicated (Colomer et al., 2008) and meta-analysis of the two studies found a significant association with time to progression (Artigalás et al., 2015). The association between rs4646 and breast cancer outcome was not replicated in other studies but this is not unexpected given that different clinical endpoints, treatment strategies and settings were used. In the context of a potential biological mechanism, the A allele of rs4646 (associated with longer overall survival) is correlated with the C allele of rs727479 (associated with lower estradiol levels). Thus, women carrying this variation would be expected to have lower levels of aromatase, enabling greater suppression of aromatase activity by AI therapy with the resultant improved patient outcomes.

It is apparent that there are other limitations to the available studies which include small sample sizes that may not have sufficient statistical power to detect associations; the genotyping of only candidate variants rather than the use of tagging SNPs, fine-mapping or imputation approaches; and the lack of adjustment for patient or clinical characteristics for all associations tested. One patient characteristic that does not appear to have been accounted for in any of the multivariate analyses of patient outcome is body mass index (BMI). There is evidence suggesting estrogen levels may not be fully suppressed by aromatase inhibitors in women with higher BMI (Folkerd et al., 2012). In future studies, BMI and all other relevant factors should be assessed for effects on clinical endpoints before inclusion in multivariate analysis models.

Some of the above limitations may soon be overcome by the Breast Cancer Stratification (B-CAST) project (http://www.b-cast.eu/). B-CAST has been established through the Breast Cancer Association Consortium (http://bcac.ccge.medschl.cam.ac.uk/bcacdata/) which incorporates clinical and genotype data from over 100,000 breast cancer patients. One of the goals of B-CAST is to understand how genetics affects clinical outcome and with the large numbers of patients available it should be possible to perform well-powered studies to comprehensively examine the effects on genetic variation on aromatase inhibitor outcome.

The use of AIs in the treatment of endometrial cancer is not well established despite the pleiotropic effect of *CYP19A1* on endometrial cancer risk (Thompson et al., 2016). Trials are ongoing for the treatment of early stage disease while response rates are low for advanced disease (Rose et al., 2000; Ma et al., 2004; Lindemann et al., 2014). The identification of *CYP19A1* variants that associate with breast cancer patient outcome would provide a strong rationale for AI treatment of endometrial cancer patients carrying these variants.

In conclusion, there is preliminary evidence that *CYP19A1* variation affects the outcomes of women treated with AIs. However, there is an urgent need for the systematic assessment of C*YP19A1* variation, in large well phenotyped clinical studies, to determine whether the genetic variants that regulate aromatase expression or activity can predict patient responses to AIs.

## Author contributions

Conception or design of the work; the acquisition, analysis and interpretation of data for the work (DG, TO, JS, and AS). Drafting the work or revising it critically for important intellectual content (DG, TO, JS, and AS). Final approval of the version to be published (DG, TO, JS, and AS). Agreement to be accountable for all aspects of the work in ensuring that questions related to the accuracy or integrity of any part of the work are appropriately investigated and resolved (DG, TO, JS, and AS).

## Funding

This work was supported by National Health and Medical Research Council of Australia Early Career Fellowship (TO; APP1111246), Senior Research Fellowship (AS; APP1061779) and project grant funding (AS; APP1109286); and a QIMR Berghofer Medical Research Institute Ph.D. scholarship (JS).

### Conflict of interest statement

The authors declare that the research was conducted in the absence of any commercial or financial relationships that could be construed as a potential conflict of interest.
